# Purification and characterization of a surfactin-like molecule produced by *Bacillus* sp. H2O-1 and its antagonistic effect against sulfate reducing bacteria

**DOI:** 10.1186/1471-2180-12-252

**Published:** 2012-11-07

**Authors:** Elisa Korenblum, Livia Vieira de Araujo, Carolina Reis Guimarães, Lauro M de Souza, Guilherme Sassaki, Fernanda Abreu, Márcia Nitschke, Ulysses Lins, Denise Maria Guimarães Freire, Eliana Barreto-Bergter, Lucy Seldin

**Affiliations:** 1 Instituto de Microbiologia Paulo de Góes, Universidade Federal do Rio de Janeiro, Rio de Janeiro, Brasil; 2 Instituto de Química, Universidade Federal do Rio de Janeiro, Rio de Janeiro, Brasil; 3 Departamento de Bioquímica, Universidade Federal do Paraná, Curitiba, Brazil; 4 Instituto de Química de São Carlos, Universidade de São Paulo, São Paulo, Brazil; 5 Laboratório de Genética Microbiana, Instituto de Microbiologia Prof. Paulo de Góes, Universidade Federal do Rio de Janeiro, Centro de Ciências da Saúde, Bloco I, Ilha do Fundão, CEP 21941-590, Rio de Janeiro, RJ, Brasil

**Keywords:** Antimicrobial substance, Surfactin-like lipopeptide, *Bacillus* sp., Sulfate reducing bacteria

## Abstract

**Background:**

*Bacillus* sp. H2O-1, isolated from the connate water of a Brazilian reservoir, produces an antimicrobial substance (denoted as AMS H2O-1) that is active against sulfate reducing bacteria, which are the major bacterial group responsible for biogenic souring and biocorrosion in petroleum reservoirs. Thus, the use of AMS H2O-1 for sulfate reducing bacteria control in the petroleum industry is a promising alternative to chemical biocides. However, prior to the large-scale production of AMS H2O-1 for industrial applications, its chemical structure must be elucidated. This study also analyzed the changes in the wetting properties of different surfaces conditioned with AMS H2O-1 and demonstrated the effect of AMS H2O-1 on sulfate reducing bacteria cells.

**Results:**

A lipopeptide mixture from AMS H2O-1 was partially purified on a silica gel column and identified via mass spectrometry (ESI-MS). It comprises four major components that range in size from 1007 to 1049 Da. The lipid moiety contains linear and branched β-hydroxy fatty acids that range in length from C13 to C16. The peptide moiety contains seven amino acids identified as Glu-Leu-Leu-Val-Asp-Leu-Leu.

Transmission electron microscopy revealed cell membrane alteration of sulfate reducing bacteria after AMS H2O-1 treatment at the minimum inhibitory concentration (5 μg/ml). Cytoplasmic electron dense inclusions were observed in treated cells but not in untreated cells. AMS H2O-1 enhanced the osmosis of sulfate reducing bacteria cells and caused the leakage of the intracellular contents. In addition, contact angle measurements indicated that different surfaces conditioned by AMS H2O-1 were less hydrophobic and more electron-donor than untreated surfaces.

**Conclusion:**

AMS H2O-1 is a mixture of four surfactin-like homologues, and its biocidal activity and surfactant properties suggest that this compound may be a good candidate for sulfate reducing bacteria control. Thus, it is a potential alternative to the chemical biocides or surface coating agents currently used to prevent SRB growth in petroleum industries.

## Background

Sulfide accumulation in petroleum reservoirs is generally described as souring. Biogenic souring is usually due to the hydrogen sulfide that is produced by sulfate reducing bacteria (SRB), a diverse group of anaerobes that use sulfate as a final electron acceptor [1]. The souring process can be intensified when the petroleum reservoir is subjected to water flooding for secondary oil recovery [2]. Because seawater is often used in water flooding in offshore oil fields, sulfate amounts raise downhole and further stimulate SRB growth, resulting in increased risk of souring. The hydrogen sulfide can reach concentrations in the reservoir that may be toxic and/or explosive. Hence, a sulfate reducing bacteria control strategy is mandatory in the oil and gas industries. Biocorrosion is also a common process in reservoirs that are subjected to secondary oil recovery [2]. In order to avoid the risks associated with the injection of sea water, the water is pretreated before being injected. The treatment usually consists of deaeration and the addition of biocides. Although different strategies of sulfide production control have been developed, the most commonly used strategy is biocide dosing with inorganic substances (chlorine; ozone) or organic compounds (quaternary ammonium salts; glutaraldehyde; tetrakis hydroxymethyl phosphonium sulphate) [3]. Quaternary ammonium salts are widely used in the Brazilian petroleum industry as a continuous biocide treatment [4]. Glutaraldehyde has been extensively applied as both batch and continuous treatment to prevent sulfate reducing bacteria growth [4,5]. However, the cost and the environmental impact of using these compounds should always be considered. A cost estimation of billions of dollars per year is predicted in oil and gas production industries due to lost material and the resources required to monitor and to prevent sulfide production, including biocide treatment [6]. For these reasons, alternative sources for avoiding or limiting the production of biogenic sulfide are needed, and the identification of new antimicrobial substances that are active against sulfate reducing bacteria is an important area of research.

Many members of the genus *Bacillus* are able to produce different types of biologically active compounds [7]. Many *Bacillus* strains are well-known for their ability to produce antimicrobial substances, including bacteriocins, exoenzymes, RNA-degrading enzymes, cell wall lytic enzymes and peptide and lipopeptide antibiotics [8-13]. Some of these substances are active only against the same species or a closely related species [14], while others have a broad spectrum of activity [15,16]. A well-known lipopeptide that is produced by *Bacillus subtilis* is surfactin, a compound named for its strong interfacial activity [17]. The structure of surfactin consists of a peptide loop of seven amino acids (L-asparagine, L-leucine, glutamic acid, L-leucine, L-valine and two D-leucines) and a hydrophobic fatty acid chain with thirteen to fifteen carbons that allows surfactin to penetrate cellular membranes. Other surfactin analogues that have been described include pumilacidin [12], bacircine [18] and lichenysin [19]. Those molecules are classified as biosurfactants because of their abilities to decrease surface tension and act as emulsifying agents [20]. Biosurfactants are amphiphilic compounds [21] that can be applied in many fields that require their capacities as detergents, emulsifying agents, lubricants, foams, wetting agents or their solubilizing and phase dispersion abilities [22-24]. Most of them also exhibit antimicrobial, anti-adhesive and anti-corrosion properties [25]. These properties are desirable for control corrosion, colonization with sulfate reducing bacteria and biofilm formation in oil facilities.

In our laboratory, an antimicrobial substance produced by a petroleum reservoir bacterium, the *Bacillus* sp. H2O-1, has been previously shown to inhibit the sessile and planktonic growth of the SRB strain *Desulfovibrio alaskensis* NCIMB 13491 [26]. This antimicrobial substance was stable at a wide pH range and at a variety of temperatures. However, further detailed biochemical studies of the antimicrobial substance produced by the *Bacillus* sp. H2O-1 strain (AMS H2O-1) were necessary to evaluate its potential use in the petroleum industry.

Therefore, this study presents the taxonomic affiliation of *Bacillus* sp. H2O-1, the structure of AMS H2O-1 and its effects on sulfate reducing bacteria cells. Furthermore, the surface free energy and the hydrophilic or hydrophobic characteristics of different surfaces conditioned with the antimicrobial substance produced by strain H2O-1 were determined and compared to surfaces treated with a surfactin produced by *B*. *subtilis* ATCC 21332.

## Methods

### Microorganisms

The antimicrobial substance producer strain *Bacillus* sp. H2O-1 was originally isolated from an oil reservoir in Brazil and previously described by Korenblum et al. [11]. This strain was grown in Luria-Bertani broth (LB), pH 7.0-7.2, containing 10 g of tryptone, 5 g of yeast extract and 5 g of NaCl per liter of distilled water. The strain *Desulfovibrio alaskensis* NCIMB 13491 was used as a sulfate reducing bacteria indicator (AMS H2O-1 sensitive) and was grown at 30°C in Postgate E medium [27] purged with a N_2_ flux to achieve anaerobiosis. *Bacillus subtilis* ATCC 21332 was used to produce surfactin as described by Nitschke [28].

### Taxonomic affiliation

The bacterial strain H2O-1 was characterized by using the kit API 50CH (Apparéils et Prócédes d′Identification – bioMérieux sa, Lyon, France) as described by the manufacturer.

In addition, the 16S rRNA gene was amplified by PCR from H2O-1 genomic DNA using the universal primers 27f (5’-AGAGTTTGATCCTGGCTCAG-3’) and 1492r (5’-GGTTACCTTGTTACGACTT-3’). DNA was extracted from *Bacillus* sp. H2O-1 grown overnight at 30°C in LB broth using the ZR Fungal/Bacterial DNA MiniPrep^TM^ kit (ZYMO Research, Irvine, CA, USA) according to the manufacturer’s instructions. The full 16S rRNA gene sequencing (GenBank accession number JX575798) was carried out by the Macrogen Genomic Division, South Korea, using ABI PRISM Big Dye Terminator Cycle Sequencing technology (Applied BioSystems, Foster city, CA, USA). The sequence obtained was compared with 16S rRNA gene sequences of closely related type strains using RDP database (http://rdp.cme.msu.edu/). Alignment and phylogenetic tree construction were performed using the Tree Builder tool from RDP website.

### Isolation and purification of the lipopeptide

The *Bacillus* sp. H2O-1 was cultured in LB broth at 30°C for three days and then harvested by centrifugation at 12,500 x g for 30 min. The supernatant was adjusted to pH 2.0 with concentrated HCl and allowed to stand overnight at 4°C. The precipitate was then dissolved in 0.4 M HCl and extracted with chloroform-methanol (2:1 v/v) [29]. The mixture was shaken vigorously and then left static for phase separation. The organic phase was concentrated at reduced pressure at 40°C, yielding the crude extract containing the lipopeptide. The AMS H2O-1 lipopeptide extract was applied to a silica gel 60 column chromatography (particle size 0.063-2 mm) and eluted with chloroform-methanol 9:1 v/v and methanol. The collected fractions were analyzed by thin layer chromatography (TLC) that was developed with CHCl_3_/CH_3_OH/ 2M NH_4_OH (40:10:1 v/v), and the spots were visualized with iodine and by spraying them with orcinol/H_2_SO_4_. The methanol fraction containing the partially purified lipopeptide was then analyzed by ESI-MS in the positive and negative ionization modes.

### Gas chromatography–mass spectrometry (GC-MS) of fatty acids

The lipids (1 mg) were methanolyzed in 0.5 ml of 1 M HCl-MeOH for 4 h at 100°C. The product containing the fatty acid methyl esters (FAMEs) was partitioned by adding H_2_O (0.5 ml) and extracting with 1 ml of n-hexane [30]. The MeOH/H_2_O phase was dried under N_2_ stream and was acetylated in pyridine-MeOH-Ac_2_O (1:1:4, v/v) with heating at 100°C for 60 min [31]. The samples were then analyzed using a GC-MS-ion trap detector (Varian, Saturn-2000R) with a capillary column DB-1-MS (J&W) that was 30 m x 0.25 mm x 0.25 μm in size. The chromatograph temperature was programmed to increase from 50 to 280°C at 20°C/min and was then held constant for 30 min. FAMEs were identified on the basis of their relative retention time in comparison with the standard of 3-hydroxy-hexadecanoate methyl ester (Sigma-Aldrich, SP, Brazil) and by their MS-fragmentation profile at electron ionization (EI – 70 eV).

### Electrospray ionization-mass spectrometry (ESI-MS)

The approximately 300 μg/ml suspension of lipids in MeOH–H_2_O (3:1, v/v) containing HCl at 1 mmol/l was submitted to positive and negative mass spectrometry at atmospheric pressure ionization and recorded on a triple quadrupole, Quattro LC (Waters) with N_2_ as the nebulization and desolvation gas. Offline analyses were performed with an infusion pump at a flow rate of 10 μl/min. The energies were set at 3.5 kV on the capillary and 100 V on the cone (negative mode) or at 3.5 kV and 90 V (positive mode). Tandem-MS was obtained by collision-induced dissociation-mass spectrometry (CID-MS) using argon as collision gas and a collision energy of 40 eV.

### Bioautography

In order to confirm the antimicrobial activity of the partial purified lipopeptide fraction, approximately 100 μl of the extract were applied to two thin layer chromatography (TLC) plates (10 cm × 20 cm) and developed with CHCl_3_/CH_3_OH/ 2M NH_4_OH (40:10:1 v/v). One plate was used as the reference chromatogram, and the spots were visualized with iodine and by spraying them with orcinol/H_2_SO_4_. The other one was used for bioautography in a Petri dish. A suspension (15 ml) containing 10^5^ cells/ml of *D*. *alaskensis* NCIMB 13491 was poured over the TLC plate. After solidification of the medium, the TLC plate was incubated for 7 days at 30°C in an anaerobic chamber. Clear growth inhibition zones were observed against a blackish background.

### Determination of the minimum inhibitory concentration (MIC) and minimum bactericidal concentration (MBC)

To determine the minimum concentration that the lipopeptide inhibits *D*. *alaskensis* NCIMB 13491 growth, the AMS H2O-1 lipopeptide extract obtained was used in microdilution susceptibility tests, which were carried out according to Das et al. [32]. A working solution of the AMS H2O-1 lipopeptide extract was prepared in distilled water (80 μg/ml) and sterilized by passing it through a 0.45 μm filter. This working solution was serially diluted to a lowest concentration of 1.2 μg/ml in sterile Postgate E medium in 96-well microtiter plates to determine the minimum inhibitory and the minimum bactericidal concentrations. The indicator strain *D*. *alaskensis* was grown for 7 days at 32°C in Postgate E medium; this culture was diluted to yield a final SRB inoculum of 10^5^ cells/ml. All of the controls and test concentrations were prepared as five replicates. The microtiter plates were incubated for 7 days at 32°C. The *D*. *alaskensis* growth was detected by observing the blackish color of the medium caused by iron sulfide precipitation in Postgate E medium. The minimum inhibitory concentration (MIC) was determined as the least amount of antimicrobial substance added that did not result in blackish color of the medium. To perform the minimum bactericidal concentration test, an aliquot of 10 μl of the treated and untreated cell suspensions from the MIC plate were used to inoculate fresh Postgate E medium (90 μl) and incubated for 7 days at 32°C. The minimum bactericidal concentration (MBC) was determined as the lowest concentration of antimicrobial substance that resulted in no growth of *D*. *alaskensis* indicator strain. All of the inoculation procedures and incubations were performed in an anaerobic chamber (PlasLabs Inc., USA).

### Preparation of cells for transmission electron microscopy (TEM)

Electron microscopy examination was used to study the biocidal effect of the AMS H2O-1 lipopeptide extract on *D*. *alaskensis* cells. After incubating 10^5^ bacterial cells/ml with AMS H2O-1 (at MIC, 0.5x MIC and 2x MIC) at 30°C for 24 hours, the cells were fixed overnight at 4°C in 2.5% glutaraldehyde in sodium cacodylate buffer 0.1M prepared in artificial sea water, washed in the same buffer, post-fixed in osmium tetroxide 1% in sodium cacodylate buffer 0.1M, washed again in the same buffer, dehydrated in an acetone series and embedded in Polybed 812. All of the ultra-thin sections were obtained using a Leica ultramicrotome, contrastained with uranyl acetate and lead citrate and observed with a FEIMorgagni TEM at 80 kV. The samples of the AMS H2O-1 treated cells and the untreated control samples were prepared in duplicate. The transmission electron microscopy preparation was also performed twice at different times.

### Physico-chemical properties

The following parameters were analyzed in order to compare the tensoactive properties of *Bacillus* sp. H2O-1 lipopeptide extract with the one produced by *B*. *subtilis* ATCC 21332, respectively: surface tension, interfacial tension and critical micellar concentration. These parameters were determined using the pendant drop technique (DSA 100S Goniometer, Model: OF 3210), according to Song and Springer [33]. The results were expressed as the mean value of at least ten pendant drops at 23°C and 55% relative humidity. Biosurfactant serial dilutions in water were performed and analyzed using the pendant drop technique described above to determine the critical micellar concentration [34]. The measurements were taken until the surface tension was close to the one of water.

### Analysis of conditioned surfaces

The surfaces samples were 2 cm^2^ coupons of stainless steel AISI 304, stainless steel AISI 430, carbon steel, galvanized steel and polystyrene. All of them were cleaned by immersing them in 99% ethanol (v/v), placing them in an ultrasonic bath for 10 min, rinsing them with distilled water, immersing them in a 2% aqueous solution of commercial detergent and ultrasonic cleaning them for 10 more minutes. The coupons were washed with distilled water and then sterilized at 121°C for 15 min. The cleaned coupons were then conditioned with aqueous solutions 5% (w/v) of the dried powder obtained after neutralization of AMS H2O-1 lipopeptide extract, surfactin or water (control) by immersing them in the solutions for 24 h at room temperature. The samples were then washed with water and left to dry at room temperature until further analysis.

The water, formamide and ethylene glycol drop angles were measured to determine the surface free energy and hydrophilic and hydrophobic characteristics of the metal and non-metal surfaces after they were conditioned with the AMS H2O-1 lipopeptide extract, surfactin, or water (control). The assays were performed using a Krüss DSA 100S goniometer (model: OF 3210) to measure the contact angles between the liquids and the different surfaces (stainless steel AISI 304, stainless steel AISI 430, carbon steel, galvanized steel and polystyrene). The results are expressed as the mean value of at least ten drops (10 μl) at 23°C and 55% relative humidity.

The surface free energy was calculated from the surface tension components from each known liquid obtained from the contact angle using the equation 1 [35]:

(1) $$\left(1+cos\theta \right)\gamma_{i}^{\mathrm{TOT}}=2\left[\left(\sqrt{\gamma_{s}^{\mathrm{LW}}\gamma_{i}^{\mathrm{LW}}}\right)+\left(\sqrt{\gamma_{s}^{+}\gamma_{i}^{-}}\right)+\left(\sqrt{\gamma_{s}^{-}\gamma_{i}^{+}}\right)\right]$$

where: θ is the contact angle between the liquid and the surface; γ^TOT^ is the total surface free energy; γ^LW^ is the Lifshitz-van der Waals component; γ^AB^ is the Lewis acid–base property; γ^+^ and γ^-^ are the electron acceptor and donor components, respectively; $\gamma^{\text{TOT}}=\gamma^{\text{LW}}+\gamma^{\text{AB}}\;\text{and}\;\gamma^{\text{AB}}=2\sqrt{\gamma^{+}\gamma^{-}}$.

The surface hydrophobicity was determined through contact angle measurements and by the approach of Van Oss [35] and Van Oss et al. [36], which states that the degree of hydrophobicity of a material (*i*) is expressed as the free energy of the interaction between two entities of that material when immersed in water (w), ΔGiwi. If the interaction between the two entities is stronger than the interaction of each entity with water, the material is considered hydrophobic (ΔGiwi<0). Hydrophilic materials have a ΔGiw*i*>0. The results were calculated according to equation 2:

(2) $$\Delta G_{\mathrm{iWi}}=-2\left(\sqrt{\gamma_{l}^{\mathrm{LW}}-\gamma_{w}^{\mathrm{LW}}}\right)-4\left(\sqrt{\gamma_{l}^{+}-\gamma_{w}^{-}}+\sqrt{\gamma_{l}^{-}-\gamma_{w}^{+}}-\sqrt{\gamma_{l}^{+}-\gamma_{l}^{-}}-\sqrt{\gamma_{w}^{+}-\gamma_{w}^{-}}\right)$$

## Results

### Taxonomic affiliation of the *Bacillus* sp. H2O-1

**Figure 1:**
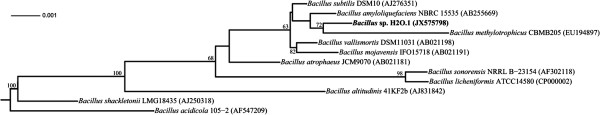
**16S rRNA gene based phylogenetic tree showing affiliation of the *Bacillus* sp.H2O-1 strain with related species of the genus *Bacillus.*** The phylogenetic tree was constructed with *Bacillus acidicola* as the outgroup using the Tree Builder algorithm of the Ribosomal Data Base Project (http://rdp.cme.msu.edu/index.jsp). Numbers at the internal nodes represent bootstrap values (> 50%). Bar = 0.001% substitutions per site.

For the determination of the phylogenetic position of strain H2O-1, its 16S rRNA gene sequence (1489 bp) was compared with those of some *Bacillus* spp. available in database. This comparison showed that strain H2O-1 was clustered in a monophyletic group together with *B*. *subtilis*, *B*. *amyloliquefaciens* and *B*. *methylotrophicus* (Figure 1). The level of 16S rRNA gene sequence similarity between H2O-1 and the type strains of *B*. *subtilis*, *B*. *amyloliquefaciens* and *B*. *methylotrophicus* were 99.8, 99.8 and 99.5%, respectively.

Strain H2O-1 was also characterized by using API 50CH test and it produced acid from glycerol, L-arabinose, ribose, D-xylose, glucose, fructose, mannose, inositol, mannitol, sorbitol, α-methyl-D-glucoside, amygdaline, arbutine, esculine, salicine, cellobiose, maltose, lactose, sucrose and trehalose. Strain H2O-1 was not able to utilize 26 other carbohydrates tested. Weak reaction was observed with melibiose, raffinose and turanose. When the API profile shown by strain H2O-1 was compared with those of the other three *Bacillus* species (*B*. *subtilis*, *B*. *amyloliquefaciens* and *B*. *methylotrophicus*), it became clear that although strain H2O-1 is very close to these *Bacillus* species it cannot be considered to represent a typical member of any one of these well-established species (Table 1). Therefore, its identification at genus level was maintained in this study.

**Table 1:** **Some biochemical characteristics that differentiate strain H2O**-**1 from reference strains of phylogenetically related *Bacillus* species**

**Characteristic**	**(1)**	**(2)**	**(3)**	**(4)**
Acid production from:				
Lactose	+	-	+	-
Inuline	-	+	-	nd
Starch	-	+	+	nd
Glycogen	-	+	-	nd
Β-gentibiose	-	+	+	nd
L-arabinose	+	+	-	+
D-xylose	+	+	-	nd
Inositol	+	+	-	+
L-rhamnose	-	-	-	+

(1) strain H2O-1; (2) *B*. *subtilis* DSM10 ^T^ (NCTC 3610 ^T^); (3) *B*. *amyloliquefaciens* NCIMB 10785 and (4) *B*. *methylotrophicus* CBMB205^T^. Data from Madhaiyan et al. [37], API 50 CH manual and this study. +, positive reaction; -, negative reaction; nd, not determined.

### Lipopeptide characterization

After being released from the lipopeptides by methanolysis, the fatty acid compositions were determined by GC-MS of the FAMEs. Five main peaks on the chromatogram were consistent with fatty acids ranging from C13 to C16. They had MS-fragmentation profile similar to that of β-hydroxy-palmitic acid methyl ester (3-OH-C16:0-*O*-Me), with a main fragment ion at *m*/*z* 103. This is derived from the cleavage of an *alpha* hydroxylated carbon (C3) that results in a diagnostic fragmentation pattern for this group that differs from the α-hydroxyl fatty acid methyl esters from a McLafferty rearrangement that result in a diagnostic fragment at *m*/*z* 90 [38]. Curiously, the chromatogram showed two main peaks that appeared close together and had retention times somewhat lower than the 3-OH-C16:0-*O*-Me. This result might be attributed to the presence of equivalent amounts of *iso*- and *anteiso*-β-OH-C15, as observed for surfactins from *Bacillus subtilis*[39].

No monosaccharides were observed in the MeOH/H_2_O phase after acetylation, indicating the absence of glycolipids. Instead, the compounds that were observed were identified as amino acids by comparison with our previous data bank [31]. The amino acids present were leucine (or isoleucine), glutamate, aspartate and valine (data not shown) and indicated a surfactin-like lipopeptide.

**Figure 2:**
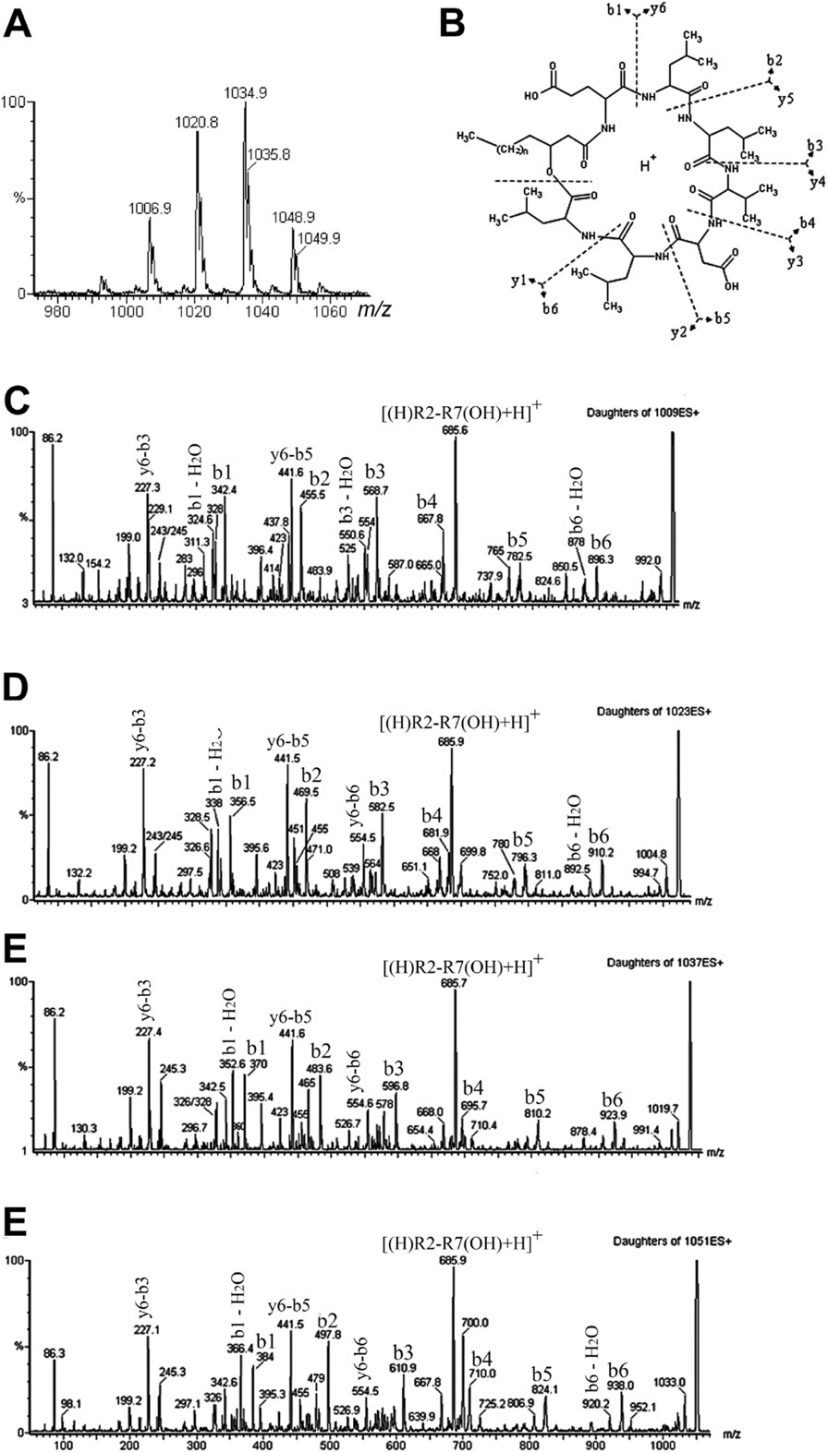
**Negative ionization mass spectrometry[M**-**H]**^**-**^**of lipopeptides(A).** The structure of the lipopeptide surfactin showing the main cleavage site on tandem-MS and the fragment nomenclature (**B**). Positive tandem MS spectra [M+H]^+^ of C13-surfactin (**C**), C14-surfactin (**D**), C15-surfactin (mixture of *iso* and *anteiso*) and C16-surfactin (**E**).

In order to confirm the lipopeptide structure, the sample was submitted to a set of ESI-MS-MS analyses. Initially, because of its anionic character (due to the presence of glutamate/aspartate), the sample was analyzed in the negative ionization MS and yielded four main ions at *m*/*z* 1007, 1021, 1035 and 1049 [M-H]^-^ (Figure 2A). These ions were consistent with the negative ions expected for surfactin with different fatty acid combinations (Figure 2B). Tandem-MS employing both of the ionization modes and with different cations or anions generally provides useful complementary information for structural analysis [40,41]. Thus, the sample was acidified (1 mM HCl) and subjected to positive ionization-MS, and ions were observed at *m*/*z* 1009, 1023, 1037 and 1051 [M+H]^+^. Therefore, the protonated lipopeptides fragmented by the CID-MS (Figures 2 C-E) revealed the same amino acid sequence as surfactin, Glu-Leu-Leu-Val-Asp-Leu-Leu, and varied only in the fatty acid moiety that was composed of β-hydroxy fatty acids of varying lengths: C13 (*m*/*z* 1009), C14 (*m*/*z* 1023), C15 (*m*/*z* 1037) and C16 (*m*/*z* 1051). This can be evidenced by the base fragment-ion, *m*/*z* 685common to every precursor-ion because it is a product of cleavages between Glu-Leu and FA-Leu, with the net charge retained in the residual hexapeptide (Leu-Leu-Val-Asp-Leu-Leu). Another abundant fragment was observed at *m*/*z* 441 and was common to every species analyzed; this fragment is a product of an y6-b5 cleavage that yields the residual tetrapeptide Leu-Leu-Val-Asp [42]. However, the fragment ions that contained the fatty acid were different by 14 mass units (m.u.) when obtained from different precursor ions. For example, the fragment b1 at *m*/*z* 370 and its dehydrated form at *m*/*z* 352 from the precursor at *m*/*z* 1037 were 14 m.u. smaller than their equivalents (*m*/*z* 384 and 366) from the precursor-ion at *m*/*z* 1051, and so on. Thus, although fragment ions from fatty acids alone were not observed, they could have been attached to the adjacent amino acids, and the overall structures were consistent with previous descriptions [42,43]. 

### Bioautography assay

**Figure 3:**
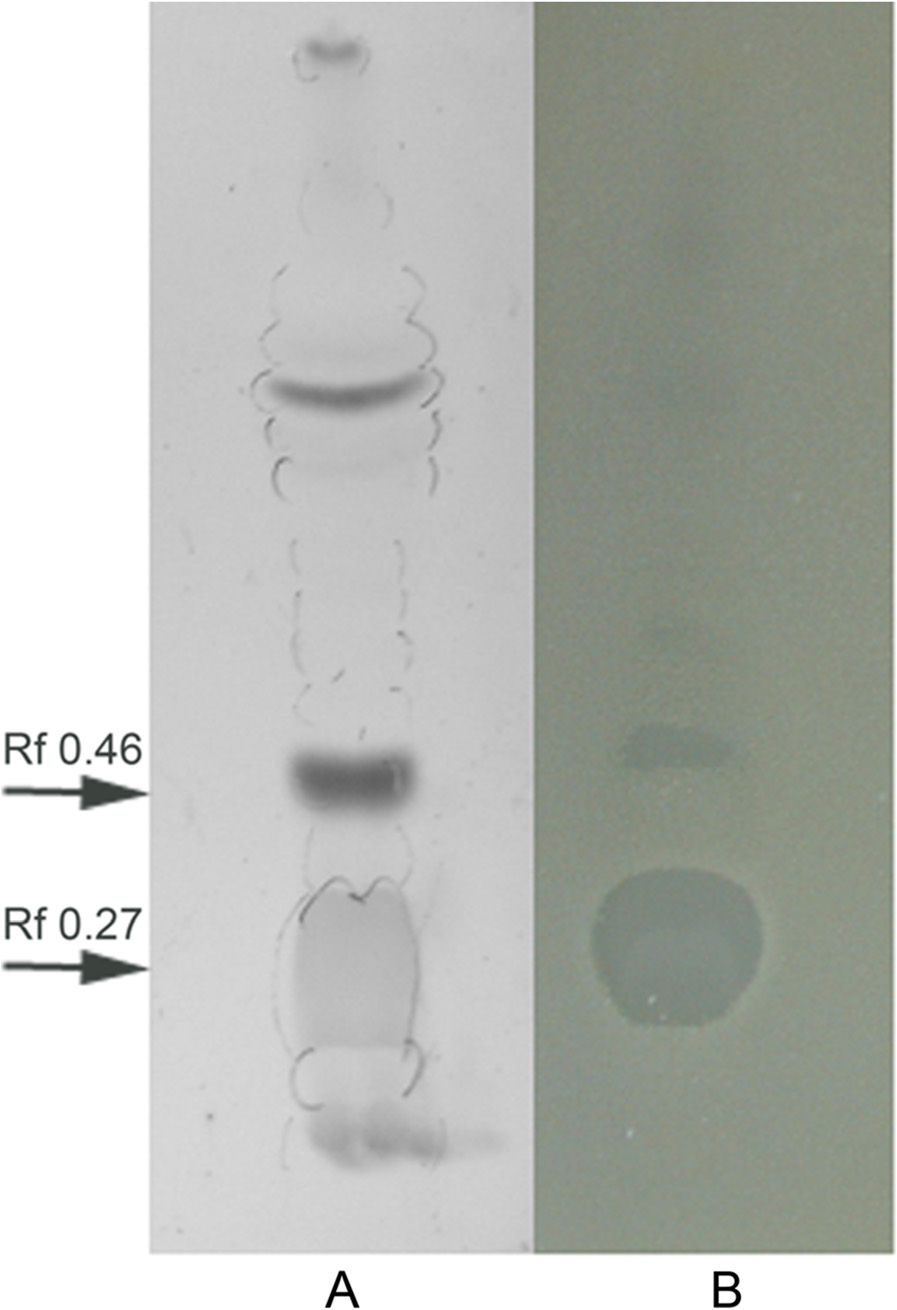
**Thin layer chromatography(TLC)analysis of the crude lipopeptide extract AMS H2O**-**1(A).** Bioautography of TLC fractions against * D *. * alaskensis * growth in an agar overlay (**B**). See text for details.

The AMS H2O-1 lipopeptide extract was analyzed by thin layer chromatography, and the separated bioactive fractions were observed in a bioautography assay (Figure 3). The compound with small R_f_ (0.27) that corresponds to the lipopeptide that was eluted from the silica gel column with methanol strongly inhibited the growth of *D*. *alaskensis*. Another compound with an R_f_ value of 0.46 that was eluted with CHCl_3_-methanol 9:1 was also active. This compound was tentatively identified as a glycolipid because it is visualized through iodine vapor and gives a violet spot with the orcinol-sulfuric acid reagent.

### Minimum inhibitory and bactericidal concentrations of AMS H2O-1 against *D*. *alaskensis* NCIMB 13491

**Figure 4:**
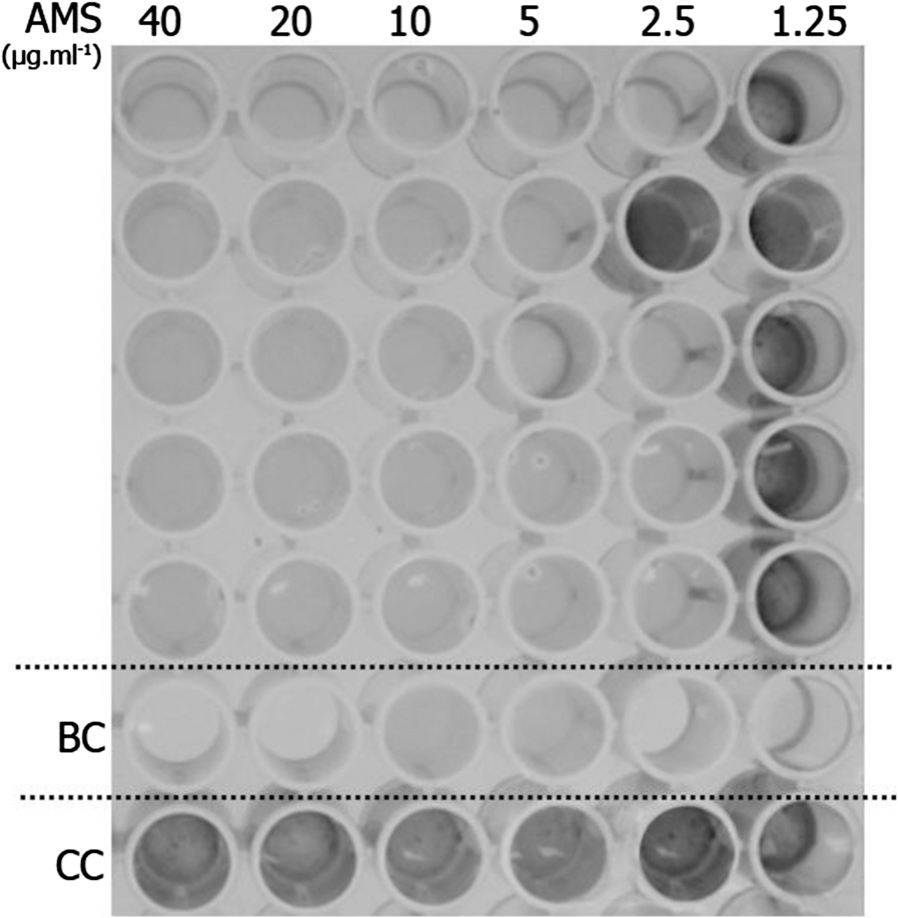
**Minimum inhibitory concentration(MIC)**) **of AMS H2O**-**1 against *D.alaskensis* NCIMB 13491 as determined by the broth microdilution method.** BC (uninoculated wells, blank medium control); CC (untreated cells, cell control).

The minimum inhibitory concentration (MIC) and the minimum bactericidal concentration (MBC) of the AMS H2O-1 lipopeptide extract were determined by the broth microdilution method using a 96 well plate. The *D*. *alaskensis* indicator strain was able to grow in contact with AMS H2O-1 at 1.5 μg/ml, as observed by the black precipitate (iron sulfide) in Postgate E medium (Figure 4). Thus, the AMS H2O-1 was able to inhibit the *D*. *alaskensis* growth at concentrations as low as 2.5 μg/ml. However, the MIC was determined to be 5 μg/ml, which was the lowest concentration that was effective against *D*. *alaskensis* in each of the five replicates (Figure 4). The minimum bactericidal concentration value of the AMS H2O-1 against *D*. *alaskensis* was established at the same value as the minimum inhibitory concentration (5 μg/ml), as no cells were recovered from any of the five replicate wells.

### Transmission electron microscopy analysis

**Figure 5:**
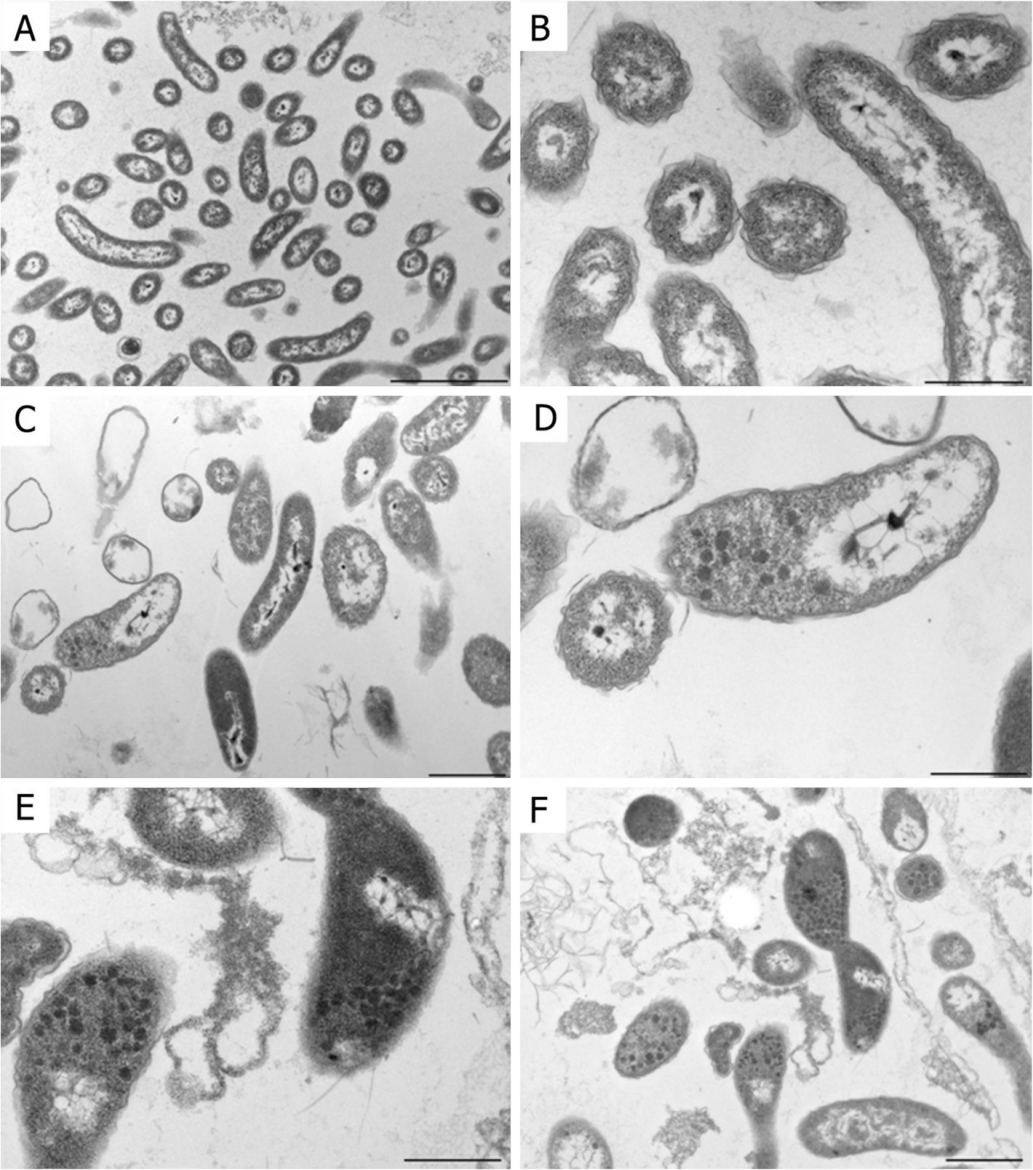
**Transmission electron microscopy micrographs of untreated *D*. *alaskensis* (A and B), after treatment with a sub-MIC level of AMS H2O-1 crude extract (C and D); and after treatment with the MIC level of AMS H2O-1 crude extract (E and F).** Bar = 3 μm (A); 1 μm (C, F); and 0.5 μm (B, D, E).

Untreated *D*. *alaskensis* cells showed normal vibrio-shaped morphology with an electron-translucent cytoplasm (Figure 5 A and B). The cell envelope was consistent with the gram-negative cell wall. Incubating the cells with a sub-MIC (0.5x MIC) concentration (2.5 μg/ml) of AMS H2O-1 lipopeptide extract resulted in cytoplasmic alterations in the form of electron-dense granules. Cytoplasm extraction was also observed in this sample, suggesting cell membrane damage (Figure 5C and D). Cells treated with the minimum inhibitory concentration (5 μg/ml) of AMS H2O-1 lipopeptide extract had increased levels of the electron dense granules, and disrupted cells were more frequently detected (Figure 5E and F).

### Physico-chemical properties

Physico-chemical analysis (Table 2) demonstrated that AMS H2O-1 lipopeptide extract is as effective as surfactin to decrease surface and interfacial tensions; both molecules achieved similar results in the applied tests. However, AMS H2O-1 showed a much lower critical micellar concentration value than the surfactin produced by *B*. *subtilis*.

**Table 2:** **Physico**-**chemical properties** (**surface tension** –**ST**, **Interfacial tension** – **IT and critical micellar concentration** – **CMC**) **of AMS H2O**-**1 and surfactin**

**Product**	**ST** (**mN**/**m**)	**IT** (**mN**/**m**)	**CMC(mg**/**L)**
**Surfactin**	26.8 ± 0.1	21.8 ± 2.8	83.7 ± 0.8
**AMS H2O**-**1**	27.1 ± 1.6	15.6 ± 1.4	27.6 ± 0.1

### Surface conditioning analysis

The results obtained from the contact angle measurements (Table 3) indicated that stainless steel AISI 304, stainless steel AISI 430, galvanized steel and polystyrene are hydrophobic according to their Δ**G**_**iwi**_ values, which classifies a surface as hydrophilic when its value is positive and hydrophobic when its value is negative. More negative values correspond to more hydrophobic surfaces, and more positive values correspond to more hydrophilic surfaces [35]. When these four surfaces were conditioned with AMS H2O-1 lipopeptide extract, they became less hydrophobic. Carbon steel (control) is hydrophilic and became hydrophobic. The surfactin treatment also decreased the hydrophobicity of some of the surfaces; all of the metal surfaces became hydrophilic with this treatment, while the polystyrene maintained the same degree of hydrophobicity. 

**Table 3:** **Energy properties of conditioned surfaces including the total surface free energy**, **the Lifshitz**-**van der Waals component**, **the Lewis acid**–**base properties**, **the electron acceptor component**, **the electron donor component and the surface hydrophobicity**

**SURFACE**/**TREATMENT**	**γLW(mJ**/**m**^**2**^**)**	**γ-(mJ**/**m**^**2**^**)**	**γ+(mJ**/**m**^**2**^**)**	**γAB(mJ**/**m**^**2**^**)**	**γTOT(mJ**/**m**^**2**^**)**	Δ**GlLw(mJ**/**m**^**2**^**)**
						
**Control**	42.02	2.68	0.85	−3.03	41	−98.7
**AMS H2O**-**1**	57.22	0.95	26.94	−10.11	47.11	−13.8
**Surfactin**	68.57	0.5	42.16	−9.19	59.39	23.7
						
**Control**	29.03	2.59	1.6	−4.07	24.96	−119.1
**AMS H2O**-**1**	47.08	0.04	14.03	−1.46	45.62	−51.0
**Surfactin**	62.71	0.63	54.11	−11.64	51.07	39.3
**CARBON STEEL**						
**Control**	75.55	2.81	40.71	−21.37	54.17	17.7
**AMS H2O**-**1**	64.68	3.5	7.68	−10.37	54.31	−81.0
**Surfactin**	71.69	1.5	49.77	−17.27	54.42	30.2
**GALVANIZED STEEL**						
**Control**	35.09	0.66	4.93	−3.61	31.48	−97.9
**AMS H2O**-**1**	16.69	1.24	43.14	−14.61	2.08	−6.8
**Surfactin**	49.71	1.72	64.89	−21.1	28.61	42.7
**POLYSTYRENE**						
**Control**	43.87	1.45	9.78	−7.53	36.34	−69.3
**AMS H2O**-**1**	62.1	1.07	18.77	−8.95	53.15	−32.1
**Surfactin**	48.01	0.37	8.96	−3.62	44.4	−70.5

The stainless steel AISI 304 and 430 and the galvanized steel donated more electrons after both treatments, while the carbon steel remained less likely to donate electrons than the control. The AMS H2O-1 treatment of the polystyrene increased its ability to donate electrons, while surfactin decreased this property.

The Lifshitz van der Waals component increased with both treatments on stainless steel 304 and 430. This component was maintained on carbon steel, galvanized steel and polystyrene with surfactin but decreased on galvanized steel and increased on polystyrene when treated with the AMS H2O-1.

The surface free energy increased on stainless steel 304 and 430 and polystyrene, was maintained on carbon steel and decreased on galvanized steel for both molecules.

## Discussion

Although synthetic surfactants are able to control corrosion and the growth of sulfate reducing bacteria, these substances may cause human and environmental health risks [44]. An alternative is the use of biosurfactants to replace the chemically synthesized surfactant compounds. Biosurfactants are biodegradable and have low toxicity [45]. The AMS H2O-1 produced by *Bacillus* sp. H2O-1 has already been shown to inhibit the growth of sulfate reducing bacteria (SRB) [11,26]. In this study, the AMS H2O-1 was characterized and was shown to have a surfactin-like lipopeptide structure. Surfactin is a biosurfactant, or an amphipathic molecule, that is a well-known product from the secondary metabolism of *B*. *subtilis*[17].

A comparative 16S rRNA gene sequence-based phylogenetic analysis placed strain H2O-1 in a clade with the species *Bacillus subtilis*, *B*. *amyloliquefaciens* and *B*. *methylotrophicus* and revealed pairwise similarities higher than 99.5%. API 50CH tests were further used to help the assignment of H2O-1 in one of these species but the fermentation of 49 sugar substances or derivatives was not sufficient for that. Therefore, the essential features for description of new taxa of the aerobic endospore-forming bacteria [46] should be used to achieve a reliable identification of strain H2O-1. In this study, this strain was considered only as a member of the genus *Bacillus* since the purification and characterization of AMS H2O-1 were the main purposes.

Different surfactin-like compounds are non-ribosomally synthesized in *Bacillus* spp., and the enzymes that are involved in those syntheses are closely related [47]. AMS H2O-1, like every surfactin-like analogue, consists of a cyclic peptide containing seven amino acid residues (mostly hydrophobic amino acids) linked to a lipidic chain. The lipophilic portion may vary in length and ramification or in the amino acid content [32]. The original surfactin molecule contains the heptapeptide sequence Glu-Leu-Leu-Val-Asp-Leu-Leu, the same found in AMS H2O-1, and a varying lipid portion of C13-C15 β-hydroxy-fatty acids that was also observed in AMS H2O-1. However, an additional lipid portion, a C16 β-hydroxy-fatty acid, was also produced by the *Bacillus* sp. H2O-1 strain under the selected conditions (shaken in a flask of LB broth at 30°C for three days). LB broth has been used in most cases for biosurfactant production from *Bacillus* strains [48]. Previous studies have shown that the length and composition of the fatty acid depends on the growth medium and may result in higher specific surfactant activity [19,49]. Regardless of the similarities between the structures of surfactin and AMS H2O-1, one of the genes required for surfactin biosynthesis, *sfp*[50], could not be detected in *Bacillus* sp. H2O-1 by PCR (data not shown) using primers previously described by Hsieh et al. [50]. These authors were able to amplify the *sfp* gene from different strains of *Bacillus subtilis* and from other surfactin-producing *Bacillus* spp. *Bacillus* sp. H2O-1 either has a mutant allele of *sfp* that could not be detected by this pair of primers or has a slightly different homologue. The expression of different homologues or different ratios of the same homologues will confer different surface tension characteristics [51].

The AMS H2O-1 lipopeptide extract was further compared with the crude extract of surfactin produced by *B*. *subtilis* for its ability to decrease interfacial tension and surface tension, and their critical micellar concentration (CMC) were determined. The results showed that the properties of both molecules were similar, although the CMC of the AMS H2O-1 lipopeptide extract was much lower (3 times), probably because of differences between the mixture of homologues produced by each species. Previous studies showed that the surfactin produced by *B*. *subtilis* LB5a using cassava waste water as substrate presented different CMC values [24,28,52].

Biosurfactants are now being widely studied because of their ability to adsorb to surfaces and delay microbial attachment. Banat et al. [20], Araujo et al. [53] and many other authors have been able to decrease microbial adhesion and biofilm development on many surfaces through the pre-treatment of the surfaces with a variety of biosurfactants. The anti-adhesive effects of a biosurfactant is due to its capacity to adsorb to a solid surface and change the hydrophobicity; the apolar portion interacts with the hydrophobic surface, while the polar portion is exposed to the aqueous environment, resulting in a decrease in the hydrophobicity of the surface. This change interferes with the microbial adhesion on this surface and therefore alters biofilm development [54]. The inhibitory activity of AMS H2O-1 on the formation of SRB biofilms on glass has been previously demonstrated [26].

Biofilm formation is a complex phenomenon that is usually divided into five steps: reversible adhesion, irreversible adhesion, EPS production, maturation and dispersion. The first and second steps involve microbial adhesion to surfaces are the most important to the initiation of biofilm formation. These steps involve physico-chemical interactions that can be mediated by non-specific interactions with long-range forces, including Lifshitz–van der Waals interactions, electrostatics, acid–base interactions, Brownian motion forces [55] and the presence or absence of cellular appendages [56]. In addition to cellular appendages, the hydrophobic interactions between the abiotic surface and the microorganism have a major role in the initial microbial adhesion and, therefore, biofilm development in biological systems [56].

Because of the ability of biosurfactants to change surface characteristics and potentially inhibit microbial adhesion and delay the corrosion of metallic surfaces [25], surfaces were conditioned with each of the biosurfactants in order to analyze their potential as a tool to control sulfate reducing bacteria and the formation of destructive biofilms in oil production facilities. The results indicated that the studied surfaces became less hydrophobic when conditioned by AMS H2O-1, with the exception of carbon steel, which became hydrophobic. Our surface hydrophobicity results agree with those of previous studies, such as the studies of Guillemot [57] and Meylheuc et al. [58], which analyzed the hydrophobic character of stainless steel conditioned with biosurfactants compared to unconditioned stainless steel (control). These authors also found that polystyrene maintained the same degree of hydrophobicity. Similar results were obtained by Araujo et al. [53], who analyzed the hydrophobic character of treated and untreated polystyrene. The anti-adhesive property of biosurfactants is due to their ability to adsorb to a surface and change its hydrophobicity according to the orientation of the molecules adsorbed; usually the apolar portion interacts with hydrophobic surfaces, and the polar portion is exposed to the aqueous environment, resulting in a decrease in the hydrophobicity of the surface [54]. When the surfaces are hydrophilic, the inverse may occur.

Stainless steel AISI 304 and 430 and galvanized steel became more electron-donating with both treatments, while carbon steel remained less electron-donating than the control. The electron-donating ability of polystyrene increased after treatment with AMS H2O-1 extract, but decreased after treatment with surfactin. Nitschke et al. [59] reported that stainless steel AISI 304 that had been conditioned with surfactin for 24 hours showed a great increase as an electron-donor and a decrease as an electron-acceptor. They concluded that surfactin modifies the surface and generates a more basic (electron-donor) surface that reduces the hydrophobicity. Our results are closely related to those found on that work, and therefore, we can state that the mixture of homologues produced by *Bacillus sp*. H2O-1 also presents these characteristics for polystyrene, stainless steel AISI 430 and galvanized steel.

Hydrophilic repulsions and hydrophobic attractions are principally due to Lewis acid–base interactions; the apolar or Lifshitz-van der Waals interactions usually only play a minor role [60]. The Lifshitz van der Waals component increased with both treatments on stainless steel 304 and 430; this component was maintained on carbon steel, galvanized steel and polystyrene with surfactin but decreased on galvanized steel and increased on polystyrene treated with the AMS H2O-1 extract. The surface free energy increased on stainless steel 304 and 430 and polystyrene, was maintained on carbon steel and decreased on galvanized steel for both molecules. These surface characteristics are strictly related to microbial adhesion and biofilm formation, and if these properties are altered by AMS H2O-1 lipopeptide extract, as demonstrated in our results, it is likely to interfere with microbial adhesion [60].

When *D*. *alaskensis* NCIMB 13491 was treated with AMS H2O-1 lipopeptide extract at the MIC (5 μg/ml), many cells with extracted cytoplasm were observed in transmission electron micrographs, and the cytoplasms of some cells were full of electron dense granules and condensed nucleoids. Although we observed cells in the micrographs after treatment, the MBC assay showed that these cells were no longer viable. The AMS H2O-1 lipopeptide extract had a bactericidal effect against the sulfate reducing bacteria tested. The surfactin-like lipopeptide critical micellar concentration (CMC) value (27.6 μg/ml) was approximately 5 times greater than the MIC (5 μg/ml), and cell shape modifications and cytoplasm electron density alterations were observed at 0.5x MIC concentration. Then, the antimicrobial effect of AMS H2O-1 is observed at concentrations lower than the CMC.

Biosurfactants in aqueous solutions form aggregates and then exhibit a lytic activity against an extensive range of microbes, possibly by forming pores and disintegrating membranes [61,62]. Sotirova and coworkers [63] observed, by scanning electron microscopy, that a biosurfactant (rhamnolipid) affects cell shape at concentrations greater than the CMC. However, Bharali and coworkers [64] observed that the rhamnolipid produced by *Pseudomonas aeruginosa* OBP1 had a CMC value of 45 μg/ml and an MIC value of 8 μg/ml against different bacteria.

Other antimicrobial compounds produced by *Bacillus* species have been tested against sulfate reducing bacteria. For example, Jayaraman et al. [65] described a peptide antibiotic produced by the gramicidin-S-overproducing *Bacillus brevis* Nagano strain that prevents sulfate reducing bacteria growth in biofilms and significantly reduced the biocorrosion of mild steel and stainless steel. The same strain has been shown to inhibit *Desulfosporosinus orientis* biofilms *in situ*[66]. The *Bacillus* strain B21, which was isolated from injection water obtained from an Algerian Sahara oilfield, was recently shown to inhibit a SRB consortium in co-culture [67] better than the biocide tetrakis hydroxymethyl phosphonium sulphate - THPS. However, the mode of action of strain B21 against sulfate reducing bacteria growth was not elucidated. Nevertheless, this growth inhibition might be due to biosurfactant production, as strain B21 was reported to produce this compound previously [68].

The use of antimicrobial substances isolated from *Bacillus* species has been of interest for SRB control in oilfields, and patents have being submitted in this field to use antimicrobials produced by *Bacillus* strains [69,70]. In order to be applied in the petroleum industry, the production of the described herein surfactin-like lipopeptide has to be optimized and scaled up, even though only a low inhibitory concentration is necessary. Because the antimicrobial lipopeptides produced by *Bacillus* generally are active against a wide range of bacteria, these molecules are also useful in the agricultural, chemical, food, and pharmaceutical industries [7,32,71]. Furthermore, in the petroleum industry, biosurfactants are important tools to assist in the biodegradation of oil spills in contaminated environments [62] and in EOR (enhanced oil recovery) or MEOR (Microbial EOR), which is a tertiary oil recovery strategy that increases petroleum yields by decreasing the surface and interfacial tensions of the oil to enable oil flow [45]. Moreover, the surfactin-like lipopeptide is produced by a bacterium that was isolated from a petroleum reservoir and could be reintroduced to the oilfield or other industrial systems in order to produce the AMS H2O-1 *in situ*.

## Conclusion

The methanol fraction of the AMS H2O-1 lipopeptide extract was analyzed by GC-MS and ESI-MS and was identified as a mixture of four surfactin-like homologues. This mixture showed excellent tensoactive properties and a lower critical micellar concentration than the surfactin produced by *B*. *subtilis*. These characteristics are of great importance for industrial applications because a lesser amount of the product is required to achieve the aim of application. The antimicrobial activity of this fraction was detected by bioautography and was observed by transmission electron microscopy. The micrographs suggested that these molecules are able to disrupt the cell walls of the strain *D*. *alaskensis* NCIMB 13491 at concentrations as low as 5 μg/ml. In addition, AMS H2O-1 surfactin-like lipopeptide has physico-chemical characteristics that are similar to those of the biosurfactant produced by *B*. *subtilis* ATCC 21332 (surfactin). Both biosurfactants adsorbed to the surface samples and changed their energy characteristics; the changes that occurred may be of great value for their ability to inhibit/decrease the initial adhesion of sulfate reducing bacteria to the surfaces. Thus, the lipopeptide biosurfactant that is produced by *Bacillus* sp. H2O-1 in this study was shown to be a potential antimicrobial biosurfactant that may be used in the petroleum industry to replace synthetic surfactants for sulfate reducing bacteria control.

## Authors’ contributions

EK, LVA, CRG, LMS, GS, and FA carried out the experiments and wrote the manuscript. MN, UL, DMG, EBB, and LS made significant revisions to the manuscript. All of the authors examined and agreed with the final manuscript.
